# Going From Zero to 100 in Remote Dementia Research: A Practical Guide

**DOI:** 10.2196/24098

**Published:** 2021-01-27

**Authors:** Megan E O'Connell, Shirin Vellani, Sheryl Robertson, Hannah M O'Rourke, Kathy S McGilton

**Affiliations:** 1 University of Saskatchewan Department of Psychology Saskatoon, SK Canada; 2 University Health Network Toronto Rehabilitation Institute University of Toronto Toronto, ON Canada; 3 University of Alberta Faculty of Nursing Edmonton, AB Canada

**Keywords:** COVID-19, telehealth, videoconferencing, dementia, information communications technology

## Abstract

Remote approaches for dementia research are required in the era of COVID-19, but moving a research program from in person to remote involves additional considerations. We recommend using outcome measures that have psychometric properties for remote delivery, and we recommend against adapting in-person scales for remote delivery without evidence for psychometric equivalency. We suggest remote research designs that maximize benefit for participants, which could have implications for control groups. Researchers should plan for flexibility in their methods for remote research and must not assume all participants will be able to videoconference; telephone-only research is possible. We recommend performing an assessment of information communication technology infrastructure and prior exposure to this technology with each participant before making a final choice on remote methods for research. In general, researchers should adapt their methods for remote research to each participant rather than requesting participants to adapt to the researchers. Screening for sensory loss should be conducted, and the impact of this on the use of technology for remote research should be considered. In this viewpoint, we detail how individualized training is required prior to engaging in remote research, how training plans interact with cognitive impairments and, finally, the steps involved in facilitating technology-based remote data collection.

## Introduction

Remote approaches for dementia research overcome barriers to participation in the era of COVID-19 requiring social distancing measures, but are also required to mitigate other factors, such as geographic barriers experienced by rural families [1-3]. In the midst of the COVID-19 pandemic, however, there are additional considerations: is this research necessary at time? Does participation involve undue stress or increase risk exposure? Does the use of remote methods undermine the quality of the research? For example, many of us are conducting remote dementia assessments, but would this method of diagnosis meet the research standards for a gold standard in validation studies? This decision-making process could have implications for research design—for example, does the use of a control group make some research untenable at this particular time given the impacts on the risk benefit analysis? Clearly, the risk benefit analysis depends on circumstances. We argue that in-person contact in the era of COVID-19 should be minimized for research, and that virtual or remote methods, which are in many situations the only option, are ethically preferred. Where an ethical decision-making process has determined the benefits to outweigh the (ideally minimal) risks, we aim in this viewpoint to provide guidance on how to move your research from in-person to remote work.

## Remote Dementia Research: Should You Do It?

Remote research can refer to research conducted solely by telephone (landline or smartphone interface) or by videoconferencing. Videoconferencing can occur via telehealth networks provided by local health care (ie, videoconferencing equipment in hospital or clinic settings) or with internet-based software platforms for participants with adequate in-home information communications technology (ICT) such as Microsoft Teams, Webex, Zoom, or Skype. The steps to consider in remote research are summarized below. We draw your attention to the ordering of some steps, which might be counterintuitive. Most notably, for those using videoconferencing for remote research, we recommend choosing a videoconferencing platform after you have conducted an ICT assessment with each participant (process described in Table 1).

**Table 1:** Zero to 100 in remote dementia research overview.

Issue	Considerations	Recommendation
1. Theoretical assessment of the feasibility of remote measurement	Are your research outcomes adaptable for remote research without compromising evidence for validity?	Choose measures with evidence for remote validity or telephone validity or choose scales with very few adaptations from in-person administration.
2. Assessment of the risks and benefits of the research design for participants	Participants may be under additional pressure and reluctant to participate if it is not clear how they will benefit. For example, is it essential to have a control group in your behavioral intervention study?	Consider participant burden as foremost to being “pandemic-friendly.” Choose a single-group repeated-measure design where individuals act as their own controls when it is unethical or not practical to have a control group, or consider use of a wait-list control group. Streamline your approach to measurement to reduce response burden.
2. Theoretical assessment of the likely ICT^a^ infrastructure of your research population	Is videoconferencing possible or is telephone-based contact most likely for remote research?	Plan for flexibility in remote research—even if you prefer videoconferencing, always include telephone-only contact as a backup plan.
3. Assessment of ICT infrastructure for each participant before research participation starts	Telephones are common, but do they have a computer, smartphone, or tablet? Do they have broadband access? Do they have speakers or headphones? Do they have a microphone?	Use a screening question like “do you have a computer, tablet, or smartphone that you use to connect with others?” A consideration: budget to send necessary ICT equipment to remote participants if appropriate.
4. Consider the needs of participants with cognitive or sensory impairments, or both	We detail special considerations for sensory and cognitive impairments, but these are highly individualized to each participant.	Screen for cognitive and sensory impairments and adapt your method of remote research accordingly.
5. Consider platform for videoconferencing research	Only some videoconferencing platforms are private and secure, which is necessary to meet REB^b^ approval. Consider what you need in terms of number of people joining and consider your participants’ experiences.	Adapt to your participant’s preferences and prior experiences with software; do not make them adapt to you.
6. Train participants for remote research	Training and support for remote research is likely required, and we detail some strategies to help with training.	Plan to spend a sizeable amount of time training participants to use new ICT equipment/platforms.
7. Obtain remote consent	We detail issues in obtaining consent, including obtaining proxy consent remotely.	The method used for remote participation should be the same as that used for informed research consent—for telephone contact, telephone consent; for videoconferenced contact, videoconferenced consent.
8. Set the scene	We detail steps required to minimize distractions during the remote visit.	Plan for communication failures by obtaining multiple methods for communication.

^a^ICT: information communications technology.

^b^REB: research ethics board.

## Remote Measurement: Can You Do It?

If you use any standardized or quantified scales for research, you must first decide if you can translate these to remote administration without invalidating the measurement properties of the scale. It is possible you will need to consider using alternative assessment methods, and it might involve changing your research design to qualitative vs quantitative. Scales that have evidence for psychometric properties under the conditions of remote administration are the best choice. It is not advisable to use a scale that has established psychometric properties only for use in person and modify this for remote administration under the assumption that it is equivalent to in-person administration. We do not recommend this practice because it introduces unknown sources of measurement error or could change the validity of measurement [4,5]. Does this mean no modified tests can be used? No, but we recommend examining the literature for evidence of impact of changing mode of administration to remote methods on measurement. We recommend following the helpful guidelines suggested by a task force on good research practices for modifying patient-reported outcomes for electronic administration [5] because they suggest levels of evidence for measurement should vary based on degree of scale modifications needed for remote administration.

Notably, Coons et al [5] recommend use of prior research to determine the levels of modifications needed to take a test from being administered in person to being remotely administered. In the event that modifications to a quantified scale for remote delivery are minor (eg, response options remain the same, but a mouse click is exchanged for circling to indicate a response), Coons et al [5] recommend cognitive debriefing. Cognitive debriefing is defined as a qualitative evaluation of how items were approached to determine if the items were understood in the way the researcher or clinician intended (ie, evidence for content validity). At the other extreme, Coons et al [5] suggest that if modifications to a test are major, which could include item wording changes or item response option changes, or a psychomotor output becomes a verbal output, it is a new test and full psychometric testing is required. Moderate modifications include wording changes and changing a non–psychomotor-based visual output to a verbal one. If modifications are moderate, this could change an item’s meaning or general content, and Coons et al [5] recommend equivalence testing. Equivalence testing can be done between and within subjects (eg, randomized cross-over designs), within subjects (eg, repeat administration in person and remote), or between subjects (eg, different groups who received the test in person vs by remote methods using differential item functioning methods or multigroup confirmatory factor analysis for measurement invariance of latent factors). If prior data for equivalency testing are not available, it might be impossible to gather these during the pandemic, which could impact your choice of measures for remote research. Irrespective of the degree of modification, normative comparison standards created for an in-person version of a measure cannot be applied to the remote-delivered version of a measure, even when the modifications for remote administration appear minor (O’Connell et al, unpublished data, 2021). Although this finding has more clinical than research implications, it impacts use of clinical scales for remote research. Ensuring the measures are reliable and valid for remote testing will involve additional pilot work prior to proceeding to the study at hand. At the very least, using scales which have not been tested remotely will need to be noted as a limitation to the findings of the study, which could jeopardize the study conclusions and undermine your remote research.

## Remote Study Designs: Are Your Modifications “Pandemic-Friendly”?

Multiple factors may complicate evaluation of interventions, particularly in situations where a randomized controlled trial is impractical, culturally or clinically unacceptable, or ethically questionable [6]. A single-group repeated-measure design can be utilized in this situation [7], which may be the case during COVID-19. In this way, the intervention is provided to all eligible participants, and the outcomes are assessed repeatedly before (thus reflecting the no-treatment condition) and after (thus reflecting the intervention condition) the intervention delivery. While there are disadvantages to this design, researchers often forget the advantages, which may be particularly relevant in a situation where participant burden is of utmost concern, such as during the current pandemic. Care home environments, for example, have been greatly impacted by COVID-19, but some research may be absolutely critical to inform how we respond to infectious disease outbreaks in ways that both saves lives and maintains quality of life. The main advantage of the single-group design is that participants serve as their own control, a situation that (1) is consistent with the counterfactual posited as ideal for determining the causal effects of an intervention [6], (2) reduces the potential of confounding as the same participants with the same personal and health or clinical characteristics are exposed to both the control and the intervention conditions [8], and (3) decreases the number of participants needed to detect significant intervention effects in the single-group repeated-measure design [9]. The number of participants needed is reduced because multiple measurements on each participant produces more data to support inferences about change. Specifically, repeated measurement of each subject (ie, individual participant data) provide enough data to adjust for baseline imbalance between treatment and control, to account for interactions among covariates, and to account for correlations between baseline and follow-up measurements of the outcome. Moreover, utilizing design approaches that do not include control groups coincide with values of inclusion and sharing of opportunity that are required as conditions for patient and community engagement [6]. Another alternative is the wait-list control group design. With a wait-list control group design, advantages include that random assignment could be maintained and that all participants would eventually receive the intervention. A disadvantage is that the data collection period is extended (to allow for outcome data collection from both intervention and control groups), and the control group may have to wait a significant amount of time to receive a potentially beneficial intervention. This may not be deemed ethical in the urgent context of COVID-19 nor be considered “pandemic-friendly.” Regardless of the design selected, the measurement approaches must be streamlined so that no participant is being asked to commit more time or energy than what is absolutely necessary to generate valid and useful knowledge that can be used to inform our response to the pandemic and beyond.

## Remote Participant Contact: Can You Do It?

This step is theoretical and will be refined in later steps, but before you engage in participant contact, consider your research participants and the likelihood of their access to the ICT infrastructure. List what ICT equipment is needed for your method of remote research and consider your participant population. Are they likely to have the ICT infrastructure? Are they likely have the knowledge to use this infrastructure without inducing undue stress? The ICT infrastructure recommended for videoconferencing includes 1024 kbps bandwidth for videoconferencing, as well as newer models of smartphones, tablets, or computers with webcam; speakers or headphones; and microphones. In our experience, this ICT infrastructure is not ubiquitous in the homes of many older adults, underscoring the lack of necessary ICT infrastructure for this population during the pandemic. Telephones are almost ubiquitous and, therefore, are a low burden method of communication. However, even their presence should not be assumed (eg, many residents of care homes do not have regular access to telephones). Our prior work detailed travel burden experienced by rural families in accessing telehealth videoconferencing [2], so in our current remote research we use telephone for intake procedures. Finally, are your planned participants likely to have sensory or cognitive impairments that make interaction with remote methods more challenging, or even impossible (we discuss this in a special section below)?

## Before You Engage in Remote Research, Assess the ICT Readiness of Each Participant

We propose initial participant contact should occur using the participant’s preferred method of contact, which for many is the telephone. Telephones are almost ubiquitous and are, therefore, a low burden method of communication (ie, they are accessible and easy to use). Before you engage in remote research, you must be in contact with each participant to assess their appropriateness/suitability for remote contact. Videoconferencing for remote research is the closest analogue to in person and, therefore, has numerous advantages [10]. Some nonverbal cues are available, visual mouth cues can help with those who have hearing loss, and rapport can be easily established when used for dementia care [1]. We discovered, however, that videoconferencing misses many nonverbal cues [2], and we recommend the researcher aims to be extremely explicit and clear in communication and be prepared to ask often for clarification of facial expressions or subtle signs of discomfort.

The goal of your ICT readiness assessment to be conducted with each participant prior to engaging in the research process is to determine if they have the necessary ICT infrastructure. Answering this deceptively simple question is made more difficult due to the bidirectional relation between ICT and participants’ comfort with and exposure to said ICT. We developed a rural technology acceptance model [3] and an Indigenous adaptation to this technology acceptance model [11], which underscore the multitude of reasons people might avoid new ICT, which includes longstanding infrastructure access barriers. If the answer to any of the following questions is no, you cannot use videoconferencing with this participant and need to consider an alternative plan such as use of the ubiquitous telephone. Recommended questions ascertain each participant’s ICT access and experience with this ICT. In our work, we see many people who would not know how to answer the question: do you have highspeed internet access? We have *not* encountered anyone able to tell us if their internet exceeds the recommended 1024 kbps bandwidth for videoconferencing. The researcher may not be able to identify or control for bandwidth issues available for participants at home; however, if the researcher has access to a bandwidth of 1024 kbps, it will ensure an acceptable quality for up to 3 connections (researcher and 2 participants) [10]. A screening question we have found useful is: do you have a computer, tablet, or smartphone that you use to connect with others? If yes, do you connect with others using video? If yes, does this video freeze and make it hard to communicate? From these questions, one can get an idea of the adequacy of their ICT infrastructure and their prior exposure in day-to-day activities. The time taken to assess the ICT infrastructure can be somewhat lengthy, but it is important to address questions to help researchers better prepare themselves and the participants. For example, this ICT infrastructure assessment may suggest the need to create a step-by-step guide, the need for specific equipment (headphones and/or webcams), the need for an informal support person to help troubleshoot with the participants, or the need to use the telephone.

## Special Consideration for Participants With Sensory Impairments

Few method exist to remotely assess sensory impairments, but a telephone-based hearing test service is available (ie, the National Hearing Test [12]) [13]. Sensory impairments could contraindicate remote methods, but this is highly individualized. For those with some hearing deficit, headphones may enhance communication since they can help with the amplification of the researcher’s voice, which is not available in face-to-face settings [10]. Severe auditory impairments can be mitigated by using closed captioning during videoconferencing, and automated and real-time closed captioning methods are available to use with some videoconferencing platforms (eg, Zoom). One will need to manipulate the videoconferencing camera placement to ensure adequate exposure to the researchers’/clinicians’ mouth-based cues, which can also mitigate hearing loss, provided, of course, the visual and auditory feed are synchronous. Headphones can help a lot with minimizing extra noise and focusing the sound, but in our experience, few participants have these at home and researchers might need to be prepared to supply these. The researcher should, however, use a system with a high-quality microphone—either a stand-alone USB microphone (situated nearer to your mouth than what would be standard on desktop computers) or a good-quality microphone on a headset. Removal of distractions can help (discussed later), but sensory challenges do not interact well with low-quality connections. Hence, we recommend considering an alternate method of remote communication if the connection is slow. Alternative methods include use of the telephone for remote research or asynchronous methods (eg, email, postal mail). Use of screens to display questionnaires can help when videoconferencing for remote research [10]; closed captioning could also be helpful for mitigating challenges in communication due to hearing loss. Additionally, we have found that visually providing a diagram with the response options to the survey questions when verbally reading them to the participant helps those with sensory or cognitive issues complete the surveys more efficiently.

## If You Choose Videoconferencing for Remote Research, Which Platform Do You Use?

We recommend that the participant should not have to adapt to you; you should adapt to them for remote research. This helps to mitigate anxiety in participants and maximize the probability of success for the remote interaction, and leverages prior learning for those with memory impairments. A final reason for using a platform your participant is familiar with is to minimize the amount of new learning required because you will need to plan to train each participant (and potentially a family member or friend who can support them) in the use of the technology platform required for your remote contact.

It might, however, not be possible to solely let your participants’ prior experiences and preferences guide your choice of software platform for videoconferencing. Foremost, the remote method platform needs to be private and secure, and if this is not possible, informed consent needs to address the potential loss of privacy or security. Local research ethics boards need to decide their comfort with nonsecure platforms for research. Many videoconferencing platforms are compatible with the Health Information Protection Act, the Personal Information Protection and Electronic Documents Act, and the Personal Health Information Protection Act, such as WebEx, Zoom Healthcare (note: not the open free version), NousTalk, Doxy.me, Microsoft Teams, and Pexip. Many of these platforms allow for group videoconferencing, which can allow a person living with dementia to join with a caregiver who may live separately. In addition, group-enabled platforms allow for live supervision of trainees.

## Remote Contact: How to Do It

### Ethical Issues

Although you might need to include the possibility of technical challenges causing frustration in your consent process, many ethical issues with remote research remain the same as for in-person research. In contrast, obtaining proxy consent when it is required in cases with diminished capacity could become more complicated under conditions of remote work. Typically for our clinical research, in-person visits are attended by someone in a position to provide proxy consent, and, as a matter of routine, we obtain assent and proxy consent when asking consent to speak with both a patient and a collateral informant or caregiver. In our diagnostic research clinic, we speak with the patient and caregiver together and separately, and we do this for our remote clinical research as well. This, however, takes multiple remote contacts sometimes spanning several days. We have not yet encountered a scenario where a live-in caregiver, such as a spousal caregiver, wishes to say something frank and their care partner will not allow them to be left to speak with us in private, but this scenario is conceivable. Attempts to time the caregiver interview during times the person living with dementia is occupied with another task might be the only method to mitigate this issue. It is also possible that a private conversation is not going to be possible, and alternate methods for private communication (ie, written) should be offered.

Despite the platform used for remote contact, you are not able to control who your participants have in their room. This has implications for privacy and confidentiality if headphones are not being used for videoconferencing or if speakerphones are used for telephone contact. We recommend a practice of introducing everyone in the room even if they are not going to be on screen or on the call.

Finally, we suggest that consent procedures should use the same procedures used for in-person contact. For in-person contact, written consent is easy and does not add to participant burden. If initial contact is over the telephone, verbal consent including its limits of confidentiality should be completed over the telephone, which can be recorded if necessary. Salmons [14] suggests that expecting the participants to download, sign, and return the consent form to the research team may “be unrealistic.” Many of the people we work with do not have computer or email access precluding the opportunity to email them a consent form, and needing to sign and return a self-addressed envelope containing a consent form would create an additional burden, never mind potentially put persons at unnecessary risk in the era of COVID-19. It is possible that research ethics boards will require a hard copy of the consent be mailed to participants so they can follow it along on the day of the consent meeting, or may require having a witness present with the participant whose name is recorded on the consent form, and then allow the consent conversation to be digitally recorded with the final step of a researcher-signed copy sent to the participants via post. This could introduce undue burden and restrict participation by some. We hope all research ethics boards consider the barriers to participation in remote service delivery and research that can be created by requiring written consent or paper-based consent processes, and the implications this has for those most vulnerable to COVID-19 and for those with limited ICT access. The research ethics board at the University of Saskatchewan, for example, has verbal consent procedures for research, which facilitates engagement in research by rural families with few ICT resources.

### Training and Troubleshooting

If you choose a method for remote contact that is not familiar to the participant or if you are asking them to use this method for remote contact in ways that are novel to them (ie, answering Likert-style questions), be prepared to spend time training them before you begin the remote research. We have detailed how independent use of videoconferencing can occur for the technologically inexperienced with remote training support [2]. We recently moved to an internet-based videoconferencing platform to engage in a socialization to mitigate isolation in the era of COVID-19, and we are prepared with training videos and solutions to common connection problems, and can screenshare to show participants how to interact with the new platform. Unfortunately, we have discovered that screenshare only works well if we are all using the same interface (Windows or Mac), computer, tablet, or smartphone. We recommend sending a step-by-step guide or having screenshots of common problems sourced from a multitude of helpful YouTube videos that you can share on a moment’s notice (a second piece of technology to source this troubleshooting information can be helpful). Regardless of your method for remote communication, we recommend planning for the worst, which could include a catastrophic failure in technology. We also advise you have a telephone contact number as a back-up and communicate how you will use this during the event of a disconnection.

It might be prudent to screen participants for cognitive impairments; numerous methods for screening for cognitive impairments have been validated for remote delivery [15,16]. Neuropsychological deficits can interact with technology use and learning of new technology; consequently, we recommend leveraging ICT methods with which the participants have prior exposure if there are cognitive impairments [17]. Persons living alone with cognitive impairment (eg, mild cognitive impairment or mild to moderate dementia or other etiologies) will require several training support sessions to engage in videoconferencing using a platform that is new to them. It is possible that persons living with moderate cognitive impairment will need remote training from an expert in cognitive rehabilitation. Common techniques from cognitive rehabilitation can train people to use new technology, even if they have marked anterograde amnesia [17], and cognitive rehabilitation can be delivered remotely to persons living with mild cognitive impairment or mild to moderate dementia [18,19]. If cognitive rehabilitation is needed to train persons living alone with mild cognitive impairment or dementia to use videoconferencing and this intervention support is not available, we recommend engaging the telehealth suites if they are available through local health care agencies or use the telephone for your remote research. Use of additional volunteers within a participant’s existing “pandemic bubble” to act as technology support can be useful and safe. For example, we spoke over the phone with a home care aid who was on site and helped with some technological challenges to facilitate the remote training needed for a participant to engage in remote research.

## Are You Ready to Go? Set the Scene

Prior to your first remote research session, it is critical that you discuss setting the scene with your participant. This brief step assumes you have had some training and practice sessions with your participant ahead of the first data collection visit if it is over videoconferencing, and you have ensured the camera angle, volume, and microphone placement are ideally set up for your encounter. You and your participant should agree on a remote contact time that is likely to be distraction-free (eg, is not during a favorite television show) and expectations regarding multitasking need to be explicit—state, “if something comes up that you have to deal with let me know and we can reschedule.” In our experience, people are less likely to multitask during videoconferencing, but we need to be more explicit in our expectations for the remote contact over the telephone. Nevertheless, telephone calls can interrupt participants during videoconferencing. Asking participants to turn off the ringer can complicate your back-up plan to use the telephone in the event of a failure in technology. We ask our participants to turn off ringers but agree on a plan to turn them back on in the event that the videoconferencing fails.

In the era of COVID-19, many clinicians and researchers are conducting remote communication from within their own homes, providing yet another venue for distractions and yet another limit of confidentiality if working from multiperson households. Examine the view your participant has of you, simplify your backdrop, and maintain a self-view lest a wayward pet comes into view and provides welcomed relief, a distraction, or both.

## Advantages Afforded by Remote Research

Remote methods for research offer several advantages beyond meeting physical distancing requirements and traversing all geographical boundaries, which reduces travel burden for participants. Foremost, research diaries of the adaptation for remote research could, in themselves, be a research output. Remote methods allow for alternative methods of participation, for example, access to language interpreters and translators could be facilitated within the remote framework. Audio and audio-video recording is seamless with many remote methods to facilitate transcription for analysis, training of staff, and supervision of staff, provided, of course, this is conducted within a secure framework and is consistent with behavioral ethics protocols and consent processes. Remote therapy will also allow for a more careful attention to treatment fidelity, which is a key methodological requirement of any sound intervention and can enhance internal validity of the trial. In a review of treatment fidelity in nonremote behavioral intervention studies, whether or not an intervention was delivered was often reported, but little attention to the other elements of fidelity such as training of staff and whether the participant actually received the intervention as intended were lacking [20]. These authors suggest that inattention to treatment fidelity may be due in part to the additional resources required to assess treatment fidelity; live supervision could mitigate some of these concerns. Live supervision conducted remotely could occur via the researcher joining a videoconference call, for example, to assess fidelity of delivery by a research assistant.

We hope to have conveyed that moving from in-person to remote dementia research is time consuming and must be completed with careful consideration, but is a worthwhile endeavor.

## Abbreviations

ICT: information communications technology
